# Perspectives and controversies surrounding strategies for institutionalizing primary health care evaluation within the Brazilian Unified National Health System

**DOI:** 10.1590/0102-311XEN165526

**Published:** 2026-11-09

**Authors:** Marly M. Cruz

**Affiliations:** 1 Fundação Oswaldo Cruz, Fiocruz, Rio de Janeiro, Brasil.

As we mark the 40th anniversary of the 8th Brazilian National Health Conference, a political and participatory milestone of the Brazilian Health Reform, we turn to the article *Evaluation in Primary Health Care in Brazil: Reflections on Institutionalization* ^1^, published by researchers from the Health Management and Evaluation Study Group of the Prof. Fernando Figueira Institute of Integral Medicine (IMIP, acronym in Portuguese). The purpose is to build on this historical-critical analysis, with an intentional, institutional, and political focus on primary health care (PHC) Monitoring and Evaluation (M&E) initiatives and the development of a policy in PHC to guide management and care. This approach provides an opportunity to reflect on its theoretical foundations and tensions, with a view to understanding the changes that have shaped evaluation practices and their use in PHC.

We thus adopt a dialogical perspective on the advances, turning points, controversies, and prospects of the process of institutionalizing PHC evaluation policy in the Brazilian Unified National Health System (SUS, acronym in Portuguese), based on a systemic and integrated approach to the strategies implemented ^2^ ^,^ ^3^ ^,^ ^4^. This is a solid and well-established experience that continues to generate knowledge, a broad research agenda, and the expansion of health teams; in other words, it provides fertile ground for different research groups and collaborative research networks ^5^ ^,^ ^6^ ^,^ ^7^.

PHC evaluation policy has driven technical and academic production, not only from a bureaucratic perspective that understand evaluation as a management function but also through integrated systems comprising critical-reflective management knowledge and practices ^8^. After all, in the face of contemporary dilemmas, it is necessary to deconstruct the technical-normative view focused on metrics and move toward a vision of evaluation that is transformative, collaborative, ethical, and transdisciplinary ^7^.

The trajectory of achievements and discontinuities helps us understand how the strategies, partnerships, and arrangements established contributed to consolidating M&E in PHC as a successful health evaluation policy experience in Brazil, despite setbacks during the period of the far-right conservative government and disputes with the private sector. As the initial policy milestone, the Primary Health Care Information System (SIAB, acronym in Portuguese) was one of the first structured information systems for systematically monitoring teams, shifting PHC management from a logic centered on administrative records to territorialized, population-based monitoring guided by indicators ^9^ ^,^ ^10^. In the same year, the Primary Health Care Indicators Pact (PIAB, acronym in Portuguese) established a framework for agreeing on indicators, targets, and responsibilities among the different levels of SUS management ^9^, changing the use of information from a primarily descriptive function to a strategic function for monitoring management ^3^.

The 2000s witnessed a vigorous expansion in the institutionalization of M&E in Brazil, driven both by internal initiatives and by international agencies, such as the World Bank, which supported the implementation of the Brazilian National Policy for Monitoring and Evaluation of PHC (PNMA/APS, acronym in Portuguese), linked to the Monitoring and Evaluation Unit and the Department of Primary Care of the Brazilian Ministry of Health ^3^ ^,^ ^9^ ^,^ ^11^. Thus, it was the Family Health Expansion and Consolidation Project (PROESF, acronym in Portuguese) that brought evaluative research, management, and care practices closer together, expanding the legitimacy of evaluation as an instrument for improving the quality of PHC and fostering an evaluative culture guided by the use of information, inter-institutional cooperation, and the production of knowledge applied to SUS management ^12^ ^,^ ^13^.

The authors ^1^ highlight the development of a sectoral evaluation policy stemming from an expanded debate within the Brazilian Ministry of Health and the Tripartite Interagency Commission, defining partnerships with academic institutions and professional associations, mobilizing institutional actors, identifying funding sources, and establishing strategies for professional training in evaluation. The authors thus emphasize how, with such guidelines, the Policy shifted evaluation from an episodic, external, or one-off practice to a strategic function of PHC management, even though its limits revealed the fragility of its sustainability as a State policy ^12^ ^,^ ^14^.

Even though innovative projects sought to overcome evaluation practices restricted to specific interventions, with the strengthening of the technical capacity of state health departments in M&E, the PROESF Baseline Studies, and the Evaluation for Quality Improvement (EQI), many questions remained regarding their effective contribution to the institutionalization of M&E. Undoubtedly, there was an expansion of cooperation and evaluative capacity between academic institutions and health services, further strengthened by the creation of the Brazilian Association of Public Health (ABRASCO, acronym in Portuguese) Thematic Group on Monitoring and Evaluation of Health Programs and Policies.

Moving forward with the Brazilian National Primary Health Care Policy (PNAB, acronym in Portuguese) and M&E strategies, the Improvement Access and Quality National Program of Primary Health Care (PMAQ-AB, acronym in Portuguese) introduced a nationwide external evaluation process and revised the self-assessment component. At the same time, it brought controversies to light by reinforcing the centrality of indicators and performance-linked financial transfers, producing tensions between improving access and quality and reducing evaluation to mechanisms of classification, certification, and financial incentives. Even so, PHC came to have a continuous process of data production capable of supporting local planning and strengthening institutional capacities, by stimulating evaluative practices among managers, health professionals, academic institutions, and services.

With the e-SUS PHC system and the citizen’s electronic health record, M&E was further strengthened through the recording of service production and longitudinal monitoring of care, with an expanded scope that favored interoperability. However, challenges of digital transformation became explicit, such as inequalities in technological infrastructure among municipalities, insufficient connectivity, low interoperability between systems, and risks of bureaucratic use of information ^6^ ^,^ ^13^ ^,^ ^15^. Thus, the potential of these systems for M&E would depend not only on the digitization of records but also on the institutional capacity to transform data into knowledge, organizational learning, social participation, and equity-oriented decisions.

With the Previne Brasil program, in the authors’ ^1^ view, there was a turning point in the institutionalization process through the reorganization of the federal financing model, based on weighted capitation, pay-for-performance, and incentives for strategic actions ^16^. Unlike PMAQ-AB, which articulated external evaluation, self-assessment, and certification, Previne Brasil placed greater weight on indicators monitored by information systems, especially those linked to individual-level recording in e-SUS PHC ^17^ ^,^ ^18^.

In light of the above, many challenges remain for the institutionalization process regarding the unequal capacity of federated entities to respond, as well as the guarantee of evaluative practices in territories marked by social, racial, economic, and environmental inequalities and unequal access to services. The authors ^1^ thus suggest that institutionalization should not be limited strictly to the formal arrangements of the health system and should consider more strategies of connection and rootedness in territories and in the relationships between State and society, incorporating dimensions related to equity, access barriers, institutional discrimination, the stigmatization of more vulnerable groups, and health inequities from a decolonial perspective ^18^.

Therefore, to deepen this debate it is necessary to raise strategic questions such as: Can evaluation effectively contribute to improving access, quality, and equity in PHC within the SUS? How can we ensure that evaluation serves as a sustainable mechanism for social transformation and the reduction of inequities? How can the lessons learned from the institutionalization of evaluation in PHC guide a digital transformation in the SUS that strengthens equity policies, social participation, and the emancipatory and transformative use of evaluation?

Ultimately, both the arguments in favor of and the criticisms directed at the different M&E strategies aim to broaden understanding of their meanings, uses, and limits. Thus, the intention is to formulate questions and propose avenues for reflection capable of mobilizing managers, health professionals, researchers, and civil society around the consolidation and broader legitimacy of evaluation practices that are more democratic, ethical, responsive, and reflexive, and that are committed to improving the quality of PHC and ensuring the universal right to health.

## Data Availability

Not applicable.
