# Supplementary material for: Perspectives and controversies surrounding strategies for institutionalizing primary health care evaluation within the Brazilian Unified National Health System
Source: Cad Saude Publica. 2026 Nov 9;42(Suppl 2):e00165526. doi: 10.1590/0102-311XEN165526 (PMC13750638; doi:10.1590/0102-311XEN165526)
Supplement: Translation

# Translation: Perspectivas e controvérsias das estratégias para a institucionalização da avaliação na atenção primária do Sistema Único de Saúde

**DOI:** 10.1590/0102-311XPT165526

**Authors:** Marly M. Cruz

**Affiliations:** 1 Fundação Oswaldo Cruz, Fiocruz, Rio de Janeiro, Brasil.

Com os 40 anos da 8ª Conferência Nacional de Saúde, como marco político e participativo da Reforma Sanitária Brasileira, voltamo-nos para o artigo *Avaliação na Atenção Primária à Saúde no Brasil: Reflexões sobre sua Institucionalização* ^1^, publicado por pesquisadores do Grupo de Estudos de Gestão e Avaliação em Saúde do Instituto de Medicina Integral Prof. Fernando Figueira (IMIP). O propósito é partir dessa análise histórico-crítica com recorte intencional, institucional e político das iniciativas de Monitoramento e Avaliação (M&A) da atenção primária à saúde (APS) e da construção de uma política neste campo que orientasse a gestão e o cuidado. Isso de forma a explorar reflexões sobre os seus fundamentos teóricos e tensões, com vistas à compreensão das mudanças que configuraram as práticas e os usos da avaliação na APS.

Parte-se assim de uma perspectiva dialógica acerca dos avanços, inflexões, controvérsias e perspectivas do processo da institucionalização da política de avaliação da APS no Sistema Único de Saúde (SUS), com base em uma abordagem sistêmica e integrada das estratégias implementadas ^2^ ^,^ ^3^ ^,^ ^4^. Uma experiência sólida e consolidada que ainda rende a produção de conhecimentos, ampla agenda de pesquisa, expansão das equipes de saúde; ou seja, solo fértil para diferentes grupos e redes colaborativas de pesquisa ^5^ ^,^ ^6^ ^,^ ^7^.

A política de avaliação da APS tem sido impulsionadora da produção técnica e acadêmica, não só baseando-se na visão burocrática de função gestora, como de sistemas integrados compostos por saberes e práticas de gestão crítico-reflexiva ^8^. Afinal, diante dos dilemas contemporâneos que são enfrentados, há que se desconstruir a visão técnico-normativa com foco nas métricas, para avançar numa visão de avaliação transformadora, colaborativa, ética e transdisciplinar ^7^.

O percurso de conquistas e descontinuidades ajudam a compreender como as estratégias, parcerias e arranjos estabelecidos contribuíram para a consolidação do M&A na APS como uma experiência bem-sucedida de política de avaliação em saúde no Brasil, apesar dos retrocessos no período de governo conservador de extrema direita e as disputas com o setor privado. Como marco inicial da política, o Sistema de Informação da Atenção Básica (SIAB) foi uma das primeiras bases informacionais estruturadas para o acompanhamento sistemático das equipes, deslocando a gestão da APS de uma lógica centrada em registros administrativos, para um monitoramento territorializado, populacional e orientado por indicadores ^9^ ^,^ ^10^. E, no mesmo ano, o Pacto de Indicadores da Atenção Básica (PIAB) estabeleceu uma dinâmica de pactuação de indicadores, metas e responsabilidades entre as esferas de gestão do SUS ^9^, alterando o uso da informação com função mais descritiva para uma função estratégica de acompanhamento da gestão ^3^.

Os anos 2000 constituíram um período efervescente de expansão da institucionalização do M&A no Brasil, induzida tanto por iniciativas internas quanto por agências internacionais, como o Banco Mundial que apoiou a implementação da Política Nacional de Monitoramento e Avaliação da APS (PNMA/APS), vinculada à Coordenação de Acompanhamento e Avaliação e Departamento de Atenção Básica do Ministério da Saúde ^3^ ^,^ ^9^ ^,^ ^11^. E, portanto, foi o Projeto de Expansão e Consolidação do Saúde da Família (PROESF) que aproximou pesquisa avaliativa, gestão e práticas assistenciais, ampliando a legitimidade da avaliação como instrumento de qualificação da APS e de indução de cultura avaliativa orientada pelo uso de informações, pela cooperação interinstitucional e pela produção de conhecimento aplicado à gestão do SUS ^12^ ^,^ ^13^.

Os autores ^1^ destacam o impulsionamento de uma política setorial de avaliação a partir de um debate ampliado no âmbito do Ministério da Saúde e da Comissão Intergestora Tripartite, definindo parcerias com instituições acadêmicas e associações profissionais, mobilização de atores institucionais, identificação de fontes de financiamento e definição de estratégias para a qualificação profissional em avaliação. Os autores ressaltam assim, com tais diretrizes, como a Política deslocou a avaliação de uma prática episódica, externa ou pontual para uma função estratégica da gestão da APS, ainda que seus limites tenham evidenciado a fragilidade de sua sustentação como política de Estado ^12^ ^,^ ^14^.

Ainda que os projetores inovadores buscassem superar as práticas avaliativas restritivas às intervenções, com o fortalecimento da capacidade técnica das secretarias estaduais de saúde em M&A, com os estudos de Linha de Base do PROESF e a Avaliação para a Melhoria da Qualidade (AMQ), muitas eram as indagações sobre a contribuição efetiva destas para a institucionalização do M&A. Pois, não há dúvida de que houve uma ampliação da cooperação e da capacidade avaliativa das instituições acadêmicas e os serviços de saúde, fortalecida ainda mais com a criação do Grupo Temático de Monitoramento e Avaliação de Programas e Políticas de Saúde da Associação Brasileira de Saúde Coletiva (ABRASCO).

Ao avançar na Política Nacional de Atenção Básica (PNAB) e nas estratégias de M&A, o Programa de Melhoria do Acesso e da Qualidade da Atenção Básica (PMAQ-AB) introduziu uma experiência nacional de avaliação externa e revisou a autoavaliação. Ao mesmo tempo, explicitou controvérsias ao reforçar a centralidade dos indicadores e o repasse financeiro vinculado ao desempenho, produzindo tensões entre a melhoria do acesso e da qualidade e a redução da avaliação a mecanismos de classificação, certificação e indução financeira. Ainda assim, a APS passou a contar com um processo contínuo de produção de dados capaz de subsidiar o planejamento local e fortalecer capacidades institucionais, ao estimular práticas avaliativas entre gestores, profissionais de saúde, instituições acadêmicas e serviços.

Com o sistema e-SUS APS e o prontuário eletrônico do cidadão se propiciou mais o M&A com o registro de produção e acompanhamento longitudinal do cuidado com a ampliação de escopo favorecendo a interoperabilidade. Contudo, explicitou-se desafios da transformação digital, como desigualdades de infraestrutura tecnológica entre municípios, conectividade insuficiente, baixa interoperabilidade entre sistemas, e riscos de uso burocrático das informações ^6^ ^,^ ^13^ ^,^ ^15^. Assim, sua potência para o M&A dependeria não apenas da digitalização dos registros, mas da capacidade institucional de transformar dados em conhecimento, aprendizagem organizacional, participação social e decisões orientadas pela equidade.

Com o programa Previne Brasil, houve, na visão dos autores ^1^, uma inflexão na institucionalização com a reorganização do modelo de financiamento federal, com base na capitação ponderada, no pagamento por desempenho e em incentivos para as ações estratégicas ^16^. Diferentemente do PMAQ-AB, que articulava avaliação externa, autoavaliação e certificação, o Previne Brasil concentrou maior peso em indicadores monitorados por sistemas de informação, especialmente vinculados ao registro individualizado no e-SUS APS ^17^ ^,^ ^18^.

Diante do exposto, ainda serão muitos os desafios para essa institucionalização no que tange a desigual capacidade de resposta dos entes federados, bem como de garantia das práticas avaliativas nos territórios marcados por desigualdades sociais, raciais, econômicas, ambientais e de acesso aos serviços. Os autores ^1^ sugerem assim que a institucionalização não deve se limitar estritamente aos arranjos formais do sistema de saúde e considerar mais estratégias de conexão e enraizamento nos territórios e nas relações entre Estado e Sociedade, incorporando dimensões relacionadas à equidade, às barreiras de acesso, às discriminações institucionais, ao estigma de grupos mais vulnerabilizados e às iniquidades em saúde numa perspectiva decolonial ^18^.

Portanto, para se aprofundar neste debate é necessário evocar questões estratégicas como: a avaliação pode contribuir efetivamente para a melhoria do acesso, da qualidade e da equidade na APS no SUS? Como garantir que a avaliação seja um dispositivo sustentável de transformação social e de redução das iniquidades? Como as lições aprendidas com a institucionalização da avaliação na APS podem orientar uma transformação digital no SUS que fortaleça as políticas de equidade, a participação social e o uso emancipatório e transformador da avaliação?

Enfim, tanto os argumentos favoráveis quanto as críticas dirigidas às diferentes estratégias de M&A, visam a ampliar a compreensão sobre seus sentidos, usos e limites. Assim, o intuito é mesmo de formular questões e propor pistas capazes de mobilizar gestores, profissionais de saúde, pesquisadores e sociedade civil em torno da consolidação e legitimidade mais ampliada de práticas avaliativas mais democráticas, éticas, responsivas, reflexivas e comprometidas com a qualificação da APS e com a garantia de direito universal à saúde.

## Data Availability

Não se aplica.
